# Sausages: Nutrition, Safety, Processing and Quality Improvement

**DOI:** 10.3390/foods10040890

**Published:** 2021-04-19

**Authors:** Javier Carballo

**Affiliations:** 1 Food Technology Area, Faculty of Sciences, University of Vigo, 32004 Ourense, Spain; carbatec@uvigo.es; 2 CITACA, Agri-Food Research and Transfer Cluster, Campus da Auga, University of Vigo, 32004 Ourense, Spain

Sausages are one of the oldest processed foods known to man. Several hundreds of varieties of sausages are produced worldwide with outstanding social and economic relevance. Each local variety within each sausage type (raw, scalded, cooked, or raw and fermented/ripened) reflects the availability of raw materials, the climate conditions of each geographical environment, the cultural and religious conditionings, and the ancestral manufacture knowledge transmitted through generations.

It is evident and expected that the different local varieties of sausages and the art of making them were perfected over centuries through the experience and the successes/failures in the production processes of the different generations.

However, similar to the manufacturing of other foods and to other production processes in general, the scientific knowledge of the physical, chemical and biological events that take place during the production processes and that are responsible for the nutritional value, sensory characteristics, and chemical and microbiological safety of the final products is relatively recent and, as in other cases, is based on the previous advances of basic disciplines such as chemistry and biology. Ultimately, it is the scientific knowledge of these events that allows them to be controlled and directed and to achieve sensory and nutritional excellence in the finished products.

Without disregarding specific works carried out in other countries, the dawn of scientific knowledge of sausage manufacturing was located in Germany during the central decades of the last 20th century. A good part of the scientific knowledge generated from the study of German local sausage varieties is collected in manuals [1,2] that are now timeless classics and an indisputable reference in the scientific literature of this field. These manuals also contain tips and indications for making sausages of high and constant quality, avoiding the most common defects and manufacturing accidents.

Among all the sausage types, the manufacture of dry-fermented sausages has special complexity, considering that the organoleptic characteristics of these products are the result of a series of modifications of the raw materials and ingredients promoted by the meat tissue enzymes and the microorganisms present, interacting with each other, and that these activities are modulated by the ingredients (salt, species, etc.) and the environmental conditions during the ripening process. As they do not receive heat treatment, they are also the sausages that present the greatest microbiological risks. The greatest research effort in this field has been devoted to the study of this type of sausages. After a series of pioneering studies that were duly reviewed by Lücke [3], starting in the 1980s of the last century studies of biochemical and microbiological characterization of the different local varieties of dry-fermented sausages multiplied. The results of all these studies, carried out mainly on local varieties of Italian, French, Spanish, Greek and Portuguese sausages obtained through spontaneous fermentation, coincide in pointing out the constancy, but uneven intensity, of the glycolytic, proteolytic, lipolytic and oxidation processes that take place in the different varieties of sausages during maturation and the role in these processes of the enzymes of muscle and fat and of the microorganisms.

Regarding the results of the studies on the microbiological characteristics of these local sausage varieties, the lactic acid bacteria and the microorganisms belonging to the *Staphylococcaceae* and *Micrococcaceae* families were revealed as the majority microbial flora with some participation of yeast and surface moulds in concrete varieties. Lactic acid bacteria develops rapidly during the fermentation of raw-cured sausages, reaching counts of 10^8^–10^9^ c.f.u./g, which remain practically stable until the end of the drying/ripening stage. The participation of this microbial group is decisive for the assurance of the hygienic/sanitary quality of the sausage, as they are responsible for the production of organic acids (lactic and acetic) and the reduction of the pH values. This decrease in pH inhibits the development of spoiling bacteria, accelerates the dehydration process of the product by reducing the water holding capacity of proteins, influences color formation and stability, and contributes to the aroma and flavor of sausages, especially those of short maturation. In addition, they can produce bacteriocins, protein compounds that also contribute to its antimicrobial activity, facilitating its implantation and inhibiting the development of unwanted microorganisms. The lipolytic activity of lactic acid bacteria is considered weak, but not so its proteolytic activity, which has been studied and demonstrated especially in different *Lactobacillus* species.

Due to their greater resistance to salt and their lower oxygen demand, staphylococci largely prevail over micrococci in this type of meat product. Staphylococci reach counts of around 10^7^ c.f.u./g. The growth of these microorganisms is inhibited by the reduction of the pH values, so that only in the case of sausages prepared with high nitrate and low carbohydrate concentrations these microorganisms can become the main microbiota outperforming lactic acid bacteria. The role of staphylococci in the manufacturing processes of meat products is mainly focused on three metabolic activities: (i) nitrate and nitrite reductase activities that make it possible to develop the typical red color in these products, as the nitric oxide formed reacts with the myoglobin and yields nitrosylmyoglobin, pink in color, (ii) catalase activity that degrades the peroxides accumulated in the sausages during the fermentation and that could have negative effects by oxidizing the iron and the cured color, also favoring lipid oxidation reactions, and (iii) proteolytic and lipolytic activities as they have numerous lipases and proteases that favors the triglyceride breakdown, and the formation of peptides, amino acids and other compounds, being all of them the origin of volatile compounds significantly affecting the aroma of the product.

Molds are aerobic micro-organisms, so their growth is fundamentally superficial, reaching in some sausage varieties counts of 10^5^–10^7^ c.f.u./cm^2^. The presence of a surface microbiota in sausages carries a series of desirable effects, prevents the formation of a superficial crust favoring homogeneous dehydration of the product, avoids rancidity by protecting the sausage from the pro-oxidant effect of light, and contributes to the development of the characteristic aroma and flavor due to the lipolytic and proteolytic capacity of some mould strains. The yeast population in raw-cured sausages has been scarcely studied because its low proportion compared to the bacterial microbiota led to the undervaluation of his role by many authors. Yeasts are found in fermented sausages at levels varying from 10^3^ c.f.u./g to 10^5^ c.f.u./g throughout the manufacturing process. The yeasts contribute to the stabilization of the color of the sausage by displacing oxygen and degrading peroxides due to its catalase activity. Furthermore, their proteolytic and lipolytic activities contribute to the development of the characteristic flavor and aroma of each product.

In the last decades, from each of these microbial groups, isolates were taken from the most common dry-fermented sausage varieties and identified using both classical and molecular methods. With some minor differences among sausage varieties, the microbial profile of the sausages obtained by spontaneous fermentations is quite consistent regardless of the types and regions of origin. Among the lactic acid bacteria, although other genera such as *Leuconostoc*, *Carnobacterium*, *Pediococcus* and *Enterococcus* have been described, the lactobacilli and above all the homofermentative lactobacilli are the main group, being *Lactobacillus sakei*, *L. curvatus*, *L. plantarum* and *L. alimentarius* in this order the main species isolated. *Lactobacillus sakei* seems to be the better adapted species to the ecosystem represented by the dry-fermented sausages and to its special environmental conditions. Among the isolates of staphylococci identified, *Staphylococcus xylosus* largely predominated followed by other species such as *S. carnosus*, *S. equorum*, *S. epidermidis*, *S. saprophyticus*, *S. lentus* and *S. sciuri*. Among the molds present in the meat and meat products, species of the genera *Mucor*, *Rhizopus*, *Aspergillus* and *Penicillium* have been described as dominant, being *P. nalgiovense* and, to a lesser extent, *P. chrysogenum* the main *Penicillium* species. *Debaryomyces hansenii* was reported as the main and more consistent species of yeast. It is the majority participation of one or the other of these microbial species together with the particular application of some processes such as smoking or the addition of specific spices and additives that configures the particularities that give personality to each of the sausages from the different countries and regions.

When raw-cured sausages are manufactured in the traditional way, without the addition of starter cultures, the prevailing environmental conditions in the sausage favor the selective growth of already adapted microbiota. As a way to ensure that this desired microbiota is present, a practice used for many years consisted of inoculating a portion of previously fermented meat to the fresh mix, with which products of greater consistency and stability are obtained. Jensen and Paddock [4] were the first authors to investigate the possibility of using a *Lactobacillus* strain in the production of raw-cured sausages, arousing the interest of other researchers, both Europeans as well as Americans, who began a more in-depth study of the starter cultures and their application to the meat industry.

The starter cultures are added to the mix in order to adequately control the fermentation and maturation processes of raw-cured sausages in such a way as to standardize the process and the quality of the end products. This is possible because the metabolic activities that the starter cultures develop during the processing of meat products and having an effect on various quality factors. The added microorganisms establish themselves as the predominant microbiota, directing fermentation and excluding the undesirable microbiota, thus reducing hygienic risks and accidents of manufacturing due to deficiencies of microbial origin. Furthermore, due to their fermentative, proteolytic and lipolytic activities, they improve the nutritional and sensory qualities of the product, while improving the speed and homogeneity of drying, which is a technological advantage. Nevertheless, commercial starter cultures must meet a number of safety requirements and must have technological competitiveness and economic viability so that their application generates the benefits expected. With regard to safety, microorganisms used as starters must not possess neither toxic nor pathogenic activity and the preparations must be free of any type of polluting, biological or chemical. With regard to technological functions, microorganisms inoculated should predominate over the spontaneous microbiota of the meat mass and develop their metabolic activity. Finally, in terms of economic aspects, the use of the starter cultures must be economically viable and easy to handle. In addition, the storage of frozen of lyophilized cultures should not affect the properties of the strain or cause losses from their activity.

The first starter culture with application in the meat industry that appeared on the market was a strain of *Pediococcus cerevisiae* [5], later classified as *Pediococcus acidilactici*, which was marketed by the Merck company in the U.S.A. in 1957 for the production of summer sausages and spreadable sausages. Almost at the same time, in Germany in 1961 it was commercialized a strain of *Micrococcus* (*Micrococcus* M53) [6] supplied by the Rudolf Müller company, and in 1966 a starter culture appeared for the first time, combining *Lactobacillus plantarum* with a strain of *Micrococcus* [7]. However, the widespread use of starter cultures in the meat industry did not begin to develop until the 1980s, mainly due to the fact that it is possible to obtain dry-fermented sausages of excellent quality without the addition of them [8]. The use of starter cultures, generally composed by a lactic acid bacteria (mainly a *Lactobacillus* strain and a coagulase-negative staphylococci (CNS), is a common and effective practice in the manufacture of fermented sausages in order to improve the color and flavor development, ensure safety and extend shelf-life.

The use of starter cultures and its effect on the microbiological, physicochemical and safety attributes of the different local varieties of dry-fermented sausages was one of the most recurrent research topic in the last decades. However, the use of a commercial non-autochthonous starter culture could have a negative impact on the sensory characteristics of the sausages, resulting in losses of the particularly desirable organoleptic properties that characterize each sausage type. Therefore, the development of specific starter cultures composed by strains isolated from spontaneous non-controlled elaborations of the corresponding type of sausage and adequately characterized in their metabolic performances has aroused the interest of researchers.

Currently the lines of research in relation to the use of starter cultures in the meat industry have been expanded in their objectives. Different authors have tried the addition of proteases and lipases in order to promote the development of the aroma and accelerate the processes of maturation in raw-cured sausages. Finally, another current line of research is on the use of probiotic lactic acid bacteria as starter cultures in raw-cured sausages, thus obtaining functional foods.

The safety of the sausages has also been the subject of abundant studies in recent decades. The microbiological hazards, mainly represented by foodborne pathogens such as *Listeria monocytogenes*, *Staphylococcus aureus*, *Escherichia coli*, *Salmonella* spp., *Yersinia enterocolitica*, and even less frequent pathogenic agents such as *Mycobacterium avium* subsp. *paratuberculosis*, *Aeromonas* spp., or hepatitis E virus, have been appropriately identified in sausages with different biochemical and technological features. In the same way, the chemical hazards, mainly biogenic amines, nitrosamines, mycotoxins, and polycyclic aromatic hydrocarbons in the particular case of smoked sausages, have been duly characterized. Measures of control and prevention of both microbiological and chemical hazards have been developed and essayed. The use of starter cultures appropriately selected can be a solution for most of these hazards.

In recent times, consumers are increasingly concerned about the relationship between health and diet, and they demand foods that are not harmful to their health and, ideally, even that protect and improve it. In this sense, there are concerns about the excessive salt content and the quantity and quality of fat in the diet and its proven relationship with some cardiovascular diseases and certain types of cancer. Traditional sausages are characterized by high salt and fat contents that are necessary to maintain their typical sensory attributes. In such scenario, the reduction of NaCl and/or its partial replacement with other salts, mainly KCl, CaCl_2_, MgCl_2_, and/or sodium ascorbate, was essayed obtaining final products having acceptable organoleptic characteristics.

Regarding the modification of the lipid fraction in sausages, the first strategies was the reduction of the fat content and/or the replacement with non-lipid substitutes. This task has not been easy because fat highly contributes to the texture, flavor and mouthfeel of these meat products. In order to reduce the fat content without compromising the organoleptic characteristics, several alternatives have been essayed consisting generally in the use of ingredients as fat substitutes and fat mimetics. In cooked meat products, some ingredients such as proteins, carbohydrates and fat-based substitutes were essayed with generally satisfactory results, especially when carbohydrates were used as fat replacers. In raw fermented sausages, the use of inulin or several other dietary fibres from fruits or cereals as fat replacers gave satisfactory results on texture and sensory properties. The use of dietary fibres as fat replacers has an added health benefit because increased proportions of fibre in the diet has been proven to reduce the risk of colon cancer, cardiovascular disease, obesity and several other disorders.

In addition to the amount of fat in the diet, the fatty acid profile of the ingested fat has a proven impact on consumer health. The formulation of the sausages has not remained unaffected by this concern. In addition to the attempt to modify the composition of animal fat used as raw material, especially through diet modifications but also by genetic selection, several studies have tried to modify the fatty acid profile of sausages by substituting animal fat by vegetable oils, mainly olive oil, or their corresponding oleogels.

The articles included in the present Special Issue show important and interesting advances and new approaches in most of the research fields mentioned.

As indicated, the study of the microbial communities of the dry-fermented sausages has been one of the most important research topics in recent years. The investigation on the microbial species diversity in fermented sausages is not only important to study the relationship between microbial species, physicochemical features and organoleptic characteristics and to gain control over quality development. It is also appropriate to better understand the link between microbial communities, specific technological features, breeding systems, geographical regions and the differential attributes that gives personality to the finished products. Comi et al. [9] studied the variability of the lactic acid bacteria in fermented sausages due to different pork breeds, breeding systems and production technology. They applied a semi-quantitative molecular method to study the alternation of the different species over time and their concentration ratios. The results of a cluster analysis in this article provide evidence of a plant-and breed-specific ecology of lactic acid bacteria. The authors have also observed that the breeding system can influence the presence of certain LAB species in the sausages.

The use of culture-independent methods presents clear advantages compared to classical methods when it comes to having a real vision of the species present and acting in a sausage throughout its production, and for this reason they have become the methods of choice in recent years. However, many of these methods still have limitations such as a taxonomic resolution not entirely satisfactory. In this context, Van Reckem et al. [10] studied the application of a previously developed high-throughput amplicon sequencing method targeting the 16S rRNA and *tuf* genes to characterize the bacterial communities of 15 fermented meats from 5 European countries. The authors also tried to associate the microbial communities of the different products with their physicochemical features and geographical origin. The obtained data broadened the view on the microbial communities associated with the products analyzed, revealing the presence of subdominant microbial species, particularly coagulase-negative *Staphylococcal* species, underreported in previous studies. On the other hand, the particular composition of the microbial communities could be linked to the special features of the particular products, mainly pH values, salt content and geographical origin.

The inclusion of a concrete microbial strain as starter culture in a fermented product requires a previous exhaustive study of its metabolic abilities in the most diverse possible environmental conditions and also in the presence of the most different substrates. All this is necessary to know what its viability and its performances are and what we can expect from that particular strain when we add it. *Lactobacillus sakei* is widely used as starter culture in fermented sausages due to its outstanding adaptation to meat environments and its ability to maintain high viability thanks to the possession of secondary pathways that are activated when hexoses are deplected. Barbieri et al. [11] studied the metabolism of *Lactobacillus sakei* Chr82, a commercial strain, in the presence of different concentrations of fermentable sugars. They inoculated this strain in a defined medium with glucose or ribose as the only carbon source, both at optimal or reduced concentrations, in order to evaluate its different metabolic and physiological responses to different growth conditions. The results evidenced different growth performances, physiological states of the cells and amino acid consumptions in relation to the carbon source and carbohydrate levels.

The probiotic bacteria have a renewed interest at the present time, given the accumulated evidence on the importance of the human intestinal microbiota on the health derived from its functionality and its interaction with the physiology of other organs, particularly skin and brain. Fermented sausages can be a good carrier for the probiotic lactic acid bacteria, and at the same time that a functional food is elaborated, the metabolic characteristics of the added strains can be exploited, making them act as starter cultures. With this aim, the use of probiotic strains as starter cultures needs a previous study on their technological characteristics to take advantage of their beneficial activities and avoid their undesirable effects on the quality and safety of sausages. Agüero et al. [12] evaluated the in vitro technological and safety characteristics in eight LAB strains having proved probiotic activity. They reported that some of them are good candidates for use as starter cultures in fermented sausages because their metabolic performances including antimicrobial activity against some of the most known pathogens. One concrete strain, *Lactobacillus rhamnosus* Lr-32, was the most promising among the tested strains but further studies are needed to check and endorse its behavior in a meat matrix.

In local varieties of fermented sausages having a strong personality and with defined and loyal markets, the use of autochthonous starter cultures is the most appropriate way to obtain manufactured products that are uniform, of high and constant quality and that retain the differentiated attributes of their homonyms artisanally obtained through spontaneous fermentations. Rodríguez-González et al. [13] studied the effect of the addition of two different autochthonous starter cultures including one strain of *Lactobacillus sakei* and one of *Staphylococcus equorum* or *Staphylococcus saprophyticus* on the biochemical changes occurring during the manufacture of Galician chorizo sausage, the most popular traditional meat product in the NW Spain. Along the manufacture of three independent batches made with each of the two starter cultures, they studied the changes in proximate composition, pH, Aw, color parameters, nitrogen fractions, free amino acids, biogenic amines, fat parameters, and free fatty acids. The two starter cultures showed good performances and seem to be suitable for increasing the quality and safety of this type of sausage.

Chemical hazards are the subject of special attention in ongoing investigations. Smoking is a process commonly applied to certain sausage types. In the past, it was applied mainly to aid in the preservation of the products due to the antimicrobial or antioxidant activity of some of the components of the smoke, and also due to a certain degree of surface dehydration that took place during the process and that contributes to the inhibition of microbial growth. At present, it is maintained as a hedonic agent, by giving the products a desirable and characteristic color, flavor and aroma. However, some of the components of smoke belonging to the family of polycyclic aromatic hydrocarbons (PAHs) are potential carcinogens and pose a chemical risk to be controlled during sausage manufacture. Usually, controlling the smoking temperature is key to limiting the deposition of PAHs during the process, but in the artisanal traditional smoking processes other strategies to achieve this end should be adopted. In the present Special Issue, Fraqueza et al. [14] make an interesting contribution in this area trying to optimize the smoking process of traditional dry-cured meat products to minimize the presence of PAHs. In this work, dry-cured sausages were submitted to four three different treatments: (i) without smoking, (ii) 20 h effective smoking, (iii) 60 h effective smoking, and (iv) effective smoking until reaching weight losses of 38–40%. The total PAHs content was generally low and did not differ significantly among the four different smoking regimes. The PAH4 (sum of four different polycyclic aromatic hydrocarbons: benzo[*a*]anthracene, chrysene, benzo[*b*]fluoranthene, and benzo[*a*]pyrene) and the benzo[*a*]pyrene contents were below the stablished legal limits in all the analyzed sausages.

The partial or total replacement of the traditional ingredients in sausages with unconventional raw materials allows the obtaining of novel and varied products capable of satisfying the needs of people who have consumption choices limited by cultural conditions, religious beliefs, personal convictions or health reasons. However, the incorporation of these new ingredients leads to the appearance of technological problems in manufacturing and also of organoleptic deficiencies in the final products that must be solved.

Within this research topic, Cullere et al. [15] studied the effect of two different fat inclusion levels, NaCl contents and LAB starter cultures on the weight loss and nutritional characteristics of an Italian-type ostrich salami. Among other results, authors observed that the lowest fat and highest NaCl level provided the greatest cumulative weight loss throughout manufacturing, but by reducing the NaCl level the weight loss was retarded without affecting the nutritional composition of the final sausage. The authors concluded that it is possible to obtain a salami from ostrich meat with lower salt and fat contents, while maintaining a satisfactory quality of the final product.

Wongkaew et al. [16] essayed the use of mango peel pectin extracted with microwave assistance as fat replacer (0%, 5%, 10% and 15% of fat replacement) in a dried Chinese sausage. Microwave-assisted extraction allows the obtaining of mango peel pectin with improved properties compared to that obtained using the conventional extraction method. Quality attributes (color, texture, and sensory characteristics) of experimental sausages were assessed and compared to those manufactured using the control formula without fat replacement. The authors concluded that the substitution of 5% of fat with pectin in the Chinese sausage could enhance color while conserve the physical qualities and sensory attributes. Therefore, mango peel pectin can be utilized in the low-fat Chinese sausage formulation as a novel fat replacer.

Vargas-Ramella et al. [17] studied the effect of partial (50%) replacement of the animal fat with healthy oils (olive, canola, and soy oil emulsions immobilized in Prosella gel) on the physicochemical attributes, texture, fatty acid profile and volatile compounds of dry-fermented deer sausage. Reformulated sausages were darker, harder, and with higher pH values than control sausages made using only animal fat. Use of soy and canola oils increased the polyunsaturated fatty acid and the w-3 fatty acid contents while decreased the w-6/w-3 ratios and the saturated fatty acid contents, thus allowing the obtaining the best nutritional properties. Regarding the effect on the volatile compounds, the animal fat replacement increased the content of the total and most of the individual volatile compounds. However, the control samples presented equal or higher contents of volatile compounds derived from the lipid oxidation processes than the reformulated sausages. Sausages reformulated with vegetable oils showed higher consumer acceptance than control sausages. In conclusion, replacement of animal fat with vegetable oils, particularly soy oil, could be an excellent strategy to elaborate healthy fermented sausages.

The use of non-meat ingredients in high percentages in sausages with the aim of obtaining healthier products may affect the microbial growth and chemical stability during storage and therefore the shelf-life of the novel sausages. Preservatives should be added in order to improve the shelf-life and safety, but consumers interested in healthier meats request at the same time products without synthetic additives. The search and the essay of natural substitutes for synthetic additives is consequently a challenge for researchers. Ranucci et al. [18] studied the effects of different concentrations of a commercial mix of extracts of pomegranate and citrus on the growth of spoilage microorganisms and on the oxidation during storage in vacuum-packaged cooked sausages made from pork meat, emmer wheat, almond and hazelnut. The authors reported that the use of the mix, particularly at a concentration of 10 g/1000 g delayed the pH decrease and the lipid oxidation processes during storage. The mix also lowered the maximum growth rate of total viable bacteria, lactic acid bacteria, and psychrotrophs. The sensory analysis carried out by sausage consumers showed an increase in the shelf-life of 6 and 16 days for the sausages made by the addition of 5‰ and 10‰ of the extract mix, respectively.

This Special Issue ends with an interesting review made by Martuscelli et al. [19]. Some religious convictions and beliefs and the restrictions imposed on the food to the practitioners entail limitations on the raw materials to be used in the manufacture of meat products (animal species and the ways in which they are slaughtered), and in processing conditions. Various scientific studies were conducted until now on ritual slaughtering practices and manufacturing of meat products for Jewish and Muslim religious communities. On the other hand, the authenticity and traceability of meat is one of the priorities of halāl food certification systems in order to prevent fraudulent practices motivated by both economic and technological reasons. In their article, Martuscelli et al. [19] reviewed studies conducted on the safety, quality and analytical authentication of halāl meat products, with particular attention on salami. The authors discussed the halāl meat products and regulations in Europe, halāl salami processing (halāl raw material, preservatives, use of spices and/or plant extracts, casings, sensory profile and the hazard represented by the biogenic amines), food safety in halāl assurance, and analytical authentication of halāl meat, salami and other meat products.

Study of the sausages is an exciting task that still offers many possibilities and motivations for researchers. Given the economic importance of sausages, the complexity and variety of the physical, chemical and microbiological processes that take place during their production that make them surprising and captivating for scientists, and the fact that many of them are deeply rooted in culture and in the heritage of various countries and geographical areas, it is certain that these products will continue to be a source of attention for scientific researchers in the future.

Some sausage varieties are still hardly known in terms of their microbiological and biochemical features, or their production processes are insufficiently standardized. In other cases, there are safety or quality concerns that must be solved so that these sausages can be enjoyed to the fullest of their potential. The improvement of the sensory quality and/or the adaptation of their sensory and nutritional properties to the changes in consumer preferences and requirements, all without sacrificing the personality and differential attributes of each sausage variety, is in all cases a permanent challenge. For all these reasons, further studies and research are essential to improve and continue enjoying these privileged foods.
